# Relationship of social capital with overweight and obesity among female health care workers

**DOI:** 10.22088/cjim.10.3.281

**Published:** 2019

**Authors:** Mojgan Firouzbakht, Mohammad Esmaeil Riahi, Karimollah Hajian-Tilaki, Abbas Ebadi, Aram Tirgar, Maryam Nikpour

**Affiliations:** 1 Social Determinant of Health Research Center, Health Research Institute, Babol University of Medical Sciences, Babol, Iran; 2 Department of Social Sciences, University of Mazandaran, Babolsar, Iran; 3 Behavioral Sciences Research Center, Life style Institiute, Nursing Faculty, Baqiyatallah University of Medical Sciences, Nursing Faculty, Tehran, Iran

**Keywords:** Overweight, Obesity, Social capital, Female workers, Iran.

## Abstract

**Background:**

The epidemic of obesity has turned into a major global health challenge. Environmental and social factors such as social capital, can significantly affect obesity. This study aimed to evaluate the relationship of social capital with overweight and obesity among female health-care workers.

**Methods:**

This cross-sectional study was conducted in 2018 on 680 female health-care workers who were randomly selected from healthcare settings affiliated to Babol University of Medical Sciences, Babol, Iran. Data were collected using a demographic questionnaire and the Workplace Social Capital (WSC) questionnaire. The SPSS Version 21 was employed to analyze the data through conducting the independent-sample *t* and the chi-square tests as well as the linear and the logistic regression analyses at a significance level of less than 0.05.

**Results:**

Linear regression analysis revealed that age, marital status, satisfaction with economic status, and structural social capital were significant predictors of body mass index (P<0.05). Moreover, logistic regression analysis indicated that weak social capital increased the odds of overweight/ obesity by 1.3 times (OR=1.345; 95% CI: 0.643–2.812) and weak structural social capital significantly increased the odds of overweight /obesity by almost four times (OR=3.757; 95% CI: 1.628–8.671; P=0.002).

**Conclusion:**

Social capital, particularly structural social capital, is a significant predictor of body mass index and a protective factor against obesity among female health-care workers. Further studies are needed to determine the paths through which social capital affects obesity- and overweight-related behaviors.

Obesity is currently a major global health challenge because its global prevalence has doubled in the past two decades (1). According to the World Health Organization report, 1.9 billion people were overweight and 650 million were obese in 2016 (2). Around 300 billion of obese people are women (3). The prevalence of obesity among the women of reproductive age varies from 3.4% to 74% (4). Overweight and obesity are also major health challenges in Iran (5), particularly Mazandaran, a northern province in Iran (6-8). The prevalence of overweight and obesity in this province is 32% and 39%, respectively (9). Obesity is the first risk factor for most chronic health conditions, including hypertension, diabetes mellitus, cardiovascular disease, and malignancies, and hence, can significantly increase mortality rate (10). It also predisposes women to polycystic ovary syndrome, reduces fertility rate, and increases the risk for pregnancy complications such as thromboembolic disorders, preeclampsia, and infection (4). Obesity is a multifactorial problem. At personal level, health-related and lifestyle behaviors are significant factors behind it.

At occupational level, factors such as shift work, long working hours, unemployment, occupational stress, job insecurity, low decision-making latitude, and obligatory extra work are associated with limited leisure activities and greater risk for obesity (13, 14). At social level, the major factors behind obesity include the level of satisfaction with socioeconomic status, income level (15), and social capital (16). Social capital is one of the factors which can affect body weight and contribute to obesity. By definition, social capital refers to resources obtained through social relationships (16). It consists of trust, mutual understanding, shared values, and behaviors which connect people like a network (17) and facilitate achieving shared interests and goals (18). Its effects on health are both negative and positive (22, 23). It exerts its effects on health through different methods, particularly the informal control and the normalization of health-related behaviors, collective actions, and social support (24). Informal control can encourage both healthy behaviors and unhealthy behaviors. Collective actions can also promote health or damage it. Finally, social support can reinforce healthy behaviors or damage health (23).

Different studies have been conducted so far into the relationship of social capital and obesity. Some studies reported the protective effects of social capital on overweight and obesity (1, 23), while some others reported social capital as a risk factor for obesity (25). It seems that the relationship of social capital with obesity is affected by the kind of interpersonal relationships, norms, and beliefs in the immediate context. In social networks, individuals have shared norms and behaviors about body shape, diet, physical activity, and overweight and any behaviors which do not conform to group norms are not supported (23). A study reported that social support helped obese people follow a downward weight loss trend through adhering to dietary regimens (26). Moreover, in social networks with unhealthy behaviors, the dark side of social capital appears. However, in social networks with weak interpersonal relationships, network members have different beliefs and norms, provide each other with different information, feel greater latitude in behavior modification, and receive greater support from each other; thus, social capital in these networks has positive effects and can reduce the risk of overweight and obesity(1). Despite studies into the risk factors of overweight and obesity in Iran (5, 6, 9), no study has evaluated the effects of social capital yet. Therefore, this study was conducted to evaluate the relationship of social capital with overweight and obesity among female health-care workers. 

## Methods

**Design and participants:** This cross-sectional study was conducted in 2018 in Babol, a city in northern province, Iran. Participants were female workers in health-care centers affiliated to Babol University of Medical Sciences, Babol, Iran. Inclusion criteria were a work experience of at least one year in the study setting, no pregnancy, no history of any systemic disorder, and no specific dietary regimen. Participants were excluded if they were unwilling to stay in the study. With a confidence level of 95%, a power of 80%, an effect size of 0.25, and a probable attrition rate of 10%, sample size was calculated to be 680. Sampling was done through stratified random sampling. Initially, healthcare settings in Babol, Iran, were divided into the two main strata of hospitals and health-care centers and then, eight health-care centers and four hospitals were randomly selected from the strata. Finally, a convenience sample was selected from each center/hospital. The number of participants selected from each center/hospital was proportionate to the total number of its health-care workers. 

**Data collection**

**Social capital:** Social capital was measured using the eight-item Workplace Social Capital (WSC) questionnaire. This questionnaire includes two main domains, namely cognitive and structural social capital. The cognitive domain (items 2, 3, and 8) consists of shared values, norms, and mutual confidence at workplace. The structural domain (items 1, 4, 5, 6, and 7) refers to collective actions and workplace participation. Items are scored from 1 (“Completely disagree”) to 5 (“Completely agree”). The possible total score of the questionnaire is 8–40, with higher scores showing stronger social capital (27). The validity and reliability of the Persian version of this questionnaire were approved in an earlier study (28). As there is no cutoff score for this questionnaire (25), we used its mean score as cutoff score to divide participants, for the purpose of logistic regression analysis, into the two groups of strong and weak social capital. Therefore, scores 24 and more were considered as strong social capital and scores less than 24 as weak social capital. 

**Covariates:** Covariates for logistic regression analysis were age (Years), marital status (Single or Married), satisfaction with sociocultural status (Low, Moderate, High), shift work (Yes, No), body mass index or BMI (Normal, Overweight, Obese), physical activity (Yes, No), alcohol consumption (Yes, No), and cigarette smoking (Yes, No). Overweight and obesity were determined using BMI. Accordingly, each participant’s height was measured in the upright standing position using a tape measure and his weight was measured using a weighing scale. Then, BMI was calculated through dividing weight (kilogram) by the square of height (meter). BMI values were interpreted as the following: 18.5–25: normal; 25–30: overweight; and more than 30: obese (29). 

Physical activity was measured using metabolic equivalent task (MET). Accordingly, participants were asked to self-report the intensity and the duration of their weekly leisure physical activities. Then, the level of energy for each physical activity was calculated as MET-minute which shows that the metabolic rate of each activity is how many times greater than the resting metabolic rate. Each one MET is the energy spent at rest (30). Based on the International Physical Activity Questionnaire (IPAQ), the level of MET for each physical activity is as follows: simple walk: 3.3 MET; fast walk: 4 MET; and regular exercise or running: 8 MET (31). These values were multiplied by the weekly time of each activity and the result was reported as MET-minute, which was interpreted as the following: less than 600: low physical activity; 600–1500: moderate physical activity; and more than 1500: high physical activity (8). For the purpose of logistic regression analysis, MET-minute value was changed into a dichotomous variable as “does not have physical activity” (with a MET-minute of less than 600) and “have physical activity” (with a MET-minute of more than 600).

**Statistical analysis: **Data were analyzed through the SPSS program for Windows (Version. 21.0). The relationships of the different levels of social capital (i.e. weak and strong) with demographic variables were examined using the independent-sample *t* and the chi-square tests. Moreover, linear regression analysis was used to determine the factors affecting overweight and obesity. The independent variables in the regression model were age (Years), work experience (Years), marital status (Single, Married), physical activity (Yes, No), place of residence (Urban areas, Rural areas), and cognitive and structural social capital (Weak, Strong). For regression analysis, variables such as educational level (Associate, Bachelor’s, Master’s or higher), satisfaction with economic status (Low, Moderate, High) which had more than two levels were changed into dichotomous dummy variables and the highest level was considered as the reference. Moreover, BMI was considered as a dichotomous variable with the two levels of normal (i.e. less than 25) and overweight/obese (i.e. more than 25). The odds ratio (OR) of overweight/obesity was calculated using logistic regression analysis. The dependent variable in the model was dichotomous BMI. The level of significance was set at less than 0.05. 

**Ethical considerations:** The Ethics Committee of Babol University of Medical Sciences, Babol, Iran, approved this study (code: MUBABOL.HRI.REC.1395.25). Written consent was obtained from all participants and they were ensured of the confidentiality of the study data and their freedom to unilaterally withdraw from the study. 

## Results

Among the 680 participants, 650 completely filled out and returned their questionnaires (response rate: 96%). The means of their age and work experience were 35.49±8.25 and 10.52±7.49 years, respectively. Table 1 shows participants’ demographic characteristics in total and based on their BMI. The mean score of participants’ social capital was 28.03±5.43 (in the range of 8–40) and 21.3% of them had weak social capital. Moreover, 51.2% of them were overweight or obese, only 22.5% of them performed physical activity, 4% smoked cigarette, and 2% consumed alcohol. BMI had significant relationships with age, work experience, marital status, educational level, shift work, and alcohol consumption (P<0.05; Table 1). 

After adjusting the effects of covariates, linear regression analysis showed that the significant predictors of BMI were age, marital status, satisfaction with economic status, and structural social capital (P<0.05; Table 2). 

Logistic regression analysis was used to determine the odds of overweight/obesity. Its results indicated that comparing with strong social capital, weak social capital increased the odds of overweight/obesity by 1.3 times (OR = 1.345; 95% CI: 0.643–2.812). Defunutely, this relationship was not statistically significant (P=0.431). Moreover, compared with strong structural social capital, weak structural social capital significantly increased the odds of overweight/obesity by almost four times (OR = 3.757; 95% CI: 1.628–8.671; P=0.002). Age was also associated with significant increase in the odds of overweight/obesity so that the odds of overweight /obesity among women younger than 35 was almost 60% of the odds among women older than 35 (OR=0.486; 95% CI: 0.343–0.689; P=0.001). Moreover, the odds of overweight/obesity among married participants was two times greater than their single counterparts (OR=2.074; 95% CI: 1.326–3.159; p=0.001) (P<0.05; Table 3).

**Table 1:** Participants’ demographic characteristics in total and in relation to their BMI

**Characteristics**		**Total** **(Mean±SD)**	**BMI**		**P value**
			**Normal** **(Mean±SD)**	**Overweight** **(Mean±SD)**	
Age (Years)		35.491±8.258	33.462±8.023	37.5±7.818	0.001
Work experience (Years)		10.52±7.497	8.948±7.108	12.12±7.572	0.001
Social capital		28.037±5.43	28.13±4.98	28.062±5.829	0.864
		N (%)	N (%)	N (%)	
Educational level	College degree	32 (4.9)	8 (2.6)	23 (7.1)	0.008
	Bachelor’s degree	536 (81.8)	253 (81.4)	267 (81.9)	
	Master’s degree or higher	87 (13.3)	50 (16.1)	36 (11)	
Marital status	Single	144 (22)	91 (29.4)	49 (15)	0.001
	Married	510 (78.0)	219 (70.6)	277 (85)	
Place of residence	Urban areas	63 (9.7)	32 (10.4)	28 (8.7)	.477
	Rural areas	586 (90.3)	277 (89.6)	294 (91.3)	
Satisfaction with economic status	High	99 (15.4)	53 (17.5)	44 (13.7)	0.302
	Moderate	468 (73.0)	219 (72.5)	237 (73.8)	
	Low	74 (11.5)	30 (9.9)	40 (12.5)	
MET-minute	< 600	496 (77.5)	223 (73.8)	257 (79.3)	0.139
	600–1500	103 (15.7)	52 (17.2)	50 (15.4)	
	< 1500	44 (6.8)	27 (8.9)	17 (5.2)	
Shift work	No	194 (29.9)	82 (26.6)	111 (34.3)	0.037
	Yes	454 (70.1)	226 (73.4)	213 (65.7)	
Cigarette smoking	No	609 (96.1)	285 (94.7)	306 (91.7)	0.122
	Yes	25 (3.9)	16 (5.3)	9 (2.9)	
Alcohol consumption	No	611 (98.1)	286 (96.3)	307 (99.7)	0.003
	Yes	12 (1.9)	11 (3.7)	1 (0.3)	

**Table 2:** The unadjusted and adjusted coefficients of linear regression analysis for the predictors of BMI

‡ **Model 2**			† **Model 1**				
**95% CI for B**	**P**	**B**	**95% CI for B**	**P**	**B**		
0.861, 1.894	0.001	0.220	0.096, 0.171	0.001	0.269	Age (Years)	
–0.656, 1.227	0.552	0.028	–0.043, 3.358	0.058	0.086	College degree	Educational level
–1.270, 2.225	0.592	0.025	_0.367, 1.500	0.234	0.054	Bachelor’s degree	
		Ref.			Ref.	Master’s degree or higher	
–2.080, –0.450	0.0092	–0.132	–2.405, –0.889	0.001	–0.167	Marital status (Married vs. Single)	
–2.440, 0.091	0.069	–0.107	–3.163, _0.655	0.003	–0.082	Low	Satisfaction with economic status
–2.355, –0.290	0.012	–0.147	_2.466, _0.412	0.006	–0.079	Moderate	
		Ref.			Ref.	High	
–1.964, 1.450	0.768	–0.013	–2.448, 0.854	0.344	–0.083	Cigarette smoking (No vs. Yes)	
–3.813, 0.961	0.241	–0.050	–4.727, –0.173	0.035	–0.086	Alcohol consumption (No vs. Yes)	
–0.838, 1.370	0.636	0.020	–0.170, 2.010	0.098	0.066	Place of residence (Urban areas vs. Rural areas)	
–0.390, –0.061	0.008	–0.199	–0.105, 0.062	0.620	–0.020	Structural social capital	
–0.021, 0.510	0.071	0.134	0.140, 0.152	0.936	0.003	Cognitive Social capital	

† Model 1. The unadjusted model

‡ Model 2: The adjusted model

**Table 3:** The unadjusted and adjusted OR in logistic regression analysis for the predictors of overweight/ obesity

‡ **Model 2**			† **Model 1**			**Characteristics**	
**95% CI for OR**	**OR**	**P**	**95% CI for OR**	**OR**	**P**		
0.343, 0.689	0.486	0.001	0.3, 0.572	0.414	0.001	Age( > 35 vs. ≤ 35)	
1.326, 3.159	2.047	0.001	1.591, 3.469	0.538	0.001	Married vs. Single	Marital status
0.755, 5.362	2.011	0.162	1.605, 9.934	3.993	0.003	College degree	Educational level
0.872, 2.443	1.459	0.150	0.924, 2.326	0.720	0.133	Bachelor’s degree	
	1	-		1	-	Master’s degree or higher	
0.485, 1.897	0.959	0.949	0.335, 1.157	0.623	0.134	High	Satisfaction with economic status
0.495, 1.599	0.858	0.585	0.488, 1.349	0.812	0.421	Moderate	
	1	-		1	-	Low	
0.845, 1.906	1.269	0.250	0.937, 1.971	1.359	0.106	Yes vs. No	Physical activity
1.628, 8.671	3.757	0.002	1.282, 4.488	2.399	0.006	Weak vs. Strong	Structural social capital
0.292, 1.040	0.551	0.066	0.730, 1.503	1.048	0.800	Weak vs. Strong	Cognitive social capital

† Model 1. The unadjusted model

‡ Model 2: The adjusted model

## Discussion

This study aimed to evaluate the relationship of social capital with overweight and obesity among female health-care workers. Findings indicated that the odds of overweight and obesity was significantly greater among women with weaker social capital. The structural domain of social capital (i.e. participation in social networks) also had significant relationship with overweight and obesity. These findings are in line with the findings of several earlier studies (1, 32-34). A study reported that the odds of obesity among women with weak social capital was 1.5 times more that women with strong social capital (15). Another study also reported that through affecting lifestyle, social capital had protective effects against obesity (35). Similarly, a study showed that strong social capital reduced the risk of obesity through promoting weight-control behaviors such as consuming low-fat diet and increasing the level of physical activity (34). Employment in healthcare settings necessitates high levels of collective actions and links together individuals from different heterogeneous social networks. In heterogeneous social networks, relationships are weak. The heterogeneity of individuals in these networks provides a wide spectrum of information and resources in different areas of health and behavior. Moreover, individuals in these networks have greater autonomy in behavioral decision-making. Therefore, membership in heterogeneous social networks can reduce the risk of obesity (1). Informal social control in these networks also increases tendency towards healthy behaviors (36) because health-related behaviors are affected by the immediate sociocultural context and social network (37, 38). For instance, the socially accepted ideal body shape in each society can significantly affect women’s behaviors towards obtaining the ideal weight; hence, fatty and muscular appearance is considered ideal and socially accepted in Latin America, while slim body is ideal and socially accepted in eastern Asian countries (23). Nonetheless, contrary to our findings, a study among Japanese employees reported that the odds of obesity was greater among male employees who had stronger social capital; this finding was attributed to high alcohol and fatty food consumption by participants in that study, particularly during weekly colleague meetings held for occupational stress management (25). In homogeneous social networks (such as families and friends), interpersonal relationships are stronger, emotional dependencies are greater, and members share similar behaviors and norms, and hence, any behavior that is different from group norms is rejected by group members (32, 39). Similarly, a study reported the significant effect of peers’ weight on motivation for weight loss (40).

A significant finding of the present study was that the prevalence of overweight and obesity among female health-care workers was as high as 51.2%. Several studies have so far reported the high prevalence of overweight and obesity among women in Iran, particularly in the northern (Mazandaran) province (5, 7, 9). This finding is attributable to the low physical activity among around 77% of our participants. Moreover, people in Mazandaran, Iran, have close familial relationships and consume high-carbohydrate, high-fat, and fried foods, all of which can result in overweight and obesity (4). They frequently attend family or peer gatherings, where they eat high-carbohydrate, high-fat, fried, and sugary foods. Similarly, a former study reported a strong relationship between social relationships and eating habits among African American women, so eating behaviors strengthened their relationships and connected them to each other in the form of family networks (37). 

Our study had some limitations. First, we could not assess all factors that could affect overweight and obesity, such as diet and eating habits; hence, the findings might have been affected to some extent by the effects of residual confounders. Second, study sample consisted only of female health-care workers and consequently, the findings may not easily be generalizable to men. Last but not the least, the study was conducted using a cross-sectional design and so, it provides no reliable data about causal relationships among study variables. Yet, as the first study into the relationship of social capital with obesity in Iran, this study had several strengths. Among the strengths of the study were its high response rate, BMI calculation through objective weight and height measurements, and social capital assessment using a standard questionnaire.

In conclusion,** t**his study indicates that female health-care workers’ strong social capital, particularly structural social capital, is associated with lower risk of obesity. In other words, social capital is a protective factor against obesity. Therefore, behavioral interventions are needed to prevent overweight and obesity among people with weak social capital. Future studies are recommended to use more comprehensive analytical strategies (such as mediation analysis and structural equation modeling) in more precisely to determine the effects of health-related behaviors and social capital on the incidence of obesity. Moreover, studies are needed to develop strategies for social capital promotion. 
